# Dealing with the unexpected: consumer responses to direct-access *BRCA* mutation testing

**DOI:** 10.7717/peerj.8

**Published:** 2013-02-12

**Authors:** Uta Francke, Cheri Dijamco, Amy K. Kiefer, Nicholas Eriksson, Bianca Moiseff, Joyce Y. Tung, Joanna L. Mountain

**Affiliations:** 1 23andMe, Inc., Mountain View, CA, USA; 2 Department of Genetics, Stanford University School of Medicine, Stanford, CA, USA

**Keywords:** BRCA mutations, Ashkenazi Jewish ancestry, Direct-access genetic testing, Direct-to-consumer genetic testing, Male BRCA carriers, Risk-reducing mastectomy, Risk-reducing oophorectomy, Cascade effect, BRCA mutation testing, Personal genomics

## Abstract

**Background.** Inherited *BRCA* gene mutations convey a high risk for breast and ovarian cancer, but current guidelines limit *BRCA* mutation testing to women with early-onset cancer and relatives of mutation-positive cases. Benefits and risks of providing this information directly to consumers are unknown.

**Methods.** To assess and quantify emotional and behavioral reactions of consumers to their 23andMe Personal Genome Service^®^ report of three *BRCA* mutations that are common in Ashkenazi Jews, we invited all 136 *BRCA*1 and *BRCA*2 mutation-positive individuals in the 23andMe customer database who had chosen to view their *BRCA* reports to participate in this IRB-approved study. We also invited 160 mutation-negative customers who were matched for age, sex and ancestry. Semi-structured phone interviews were completed for 32 mutation carriers, 16 women and 16 men, and 31 non-carriers. Questions addressed personal and family history of cancer, decision and timing of viewing the *BRCA* report, recollection of the result, emotional responses, perception of personal cancer risk, information sharing, and actions taken or planned.

**Results.** Eleven women and 14 men had received the unexpected result that they are carriers of a *BRCA*1 185delAG or 5382insC, or *BRCA*2 6174delT mutation. None of them reported extreme anxiety and four experienced moderate anxiety that was transitory. Remarkably, five women and six men described their response as neutral. Most carrier women sought medical advice and four underwent risk-reducing procedures after confirmatory mutation testing. Male carriers realized that their test results implied genetic risk for female relatives, and several of them felt considerably burdened by this fact. Sharing mutation information with family members led to screening of at least 30 relatives and identification of 13 additional carriers. Non-carriers did not report inappropriate actions, such as foregoing cancer screening. All but one of the 32 mutation-positive participants appreciated learning their *BRCA* mutation status.

**Conclusions.** Direct access to *BRCA* mutation tests, considered a model for high-risk actionable genetic tests of proven clinical utility, provided clear benefits to participants. The unexpected information demonstrated a cascade effect as relatives of newly identified carriers also sought testing and more mutation carriers were identified. Given the absence of evidence for serious emotional distress or inappropriate actions in this subset of mutation-positive customers who agreed to be interviewed for this study, broader screening of Ashkenazi Jewish women for these three BRCA mutations should be considered.

## Introduction

Direct-to-consumer (DTC) genetic health information first became available in 2007 when three companies started offering microarray-based genotyping of genome-wide single-nucleotide variants (23andMe; DeCodeMe; Navigenics). At present, health reports provided online to consumers include heterogeneous risk information based on results of published genome-wide association studies of distinct populations, carrier status for known Mendelian recessive disorders, variants affecting drug response and sensitivity to side effects, and a few rare high-impact Mendelian dominant mutations with disease-onset later in life (e.g. 23andMe, DeCODE Genetics; Pathway Genomics).

Concerns expressed in a large body of literature and position statements issued by professional societies postulate that high-impact genetic information should not be disseminated DTC because consumers will not be able to understand the meaning, or will misunderstand it; positive test results could cause panic and inappropriate actions, possibly putting undue burden on the health care system; and negative test results could cause false reassurance and inappropriate actions such as foregoing recommended cancer screening (Berliner & Fay, 2007; American College of Medical Genetics, 2008; McGuire & Burke, 2008; Annes, Giovanni & Murray, 2010; Robson et al., 2010; Skirton et al., 2012). Very few published studies have addressed the validity of these claims. Bloss and colleagues enrolled participants in a research study to assess reactions to results and subsequent actions taken, such as medical consultations and lifestyle changes (Bloss, Schork & Topol, 2011). Their study found little positive or negative effect of gaining access to this information. The panel of tests, however, was limited to low-impact single nucleotide polymorphism (SNP) associations with unclear clinical utility. There is a single case report of psychological distress in a woman who received a BRCA mutation report DTC that was relieved by genetic counseling (Dohany et al., 2012). In a survey study in which DTC genetic testing customers were asked to interpret hypothetical scenarios of type 2 diabetes and colorectal cancer risk, over 90% understood the meaning correctly (Kaufman et al., 2012).

This study focuses on reactions of individuals who received their own test results of testing for three mutations that predispose one to hereditary breast and ovarian cancer (HBOC). These mutations are most common in people with Ashkenazi Jewish (AJ) ancestry: *BRCA1* 185delAG and 5382insC, *BRCA2* 6174delT. *BRCA1* mutations confer upon women a breast cancer risk of about 60% and an ovarian cancer risk of about 40%; *BRCA2* mutations confer a breast cancer risk of about 50% and an ovarian cancer risk of about 20% (Chen & Parmigiani, 2007). Among all predictive genetic tests currently available DTC, *BRCA* mutation testing can be considered the most actionable with proven clinical utility (Domchek et al., 2010).

Growing activity in the area of whole genome and exome sequencing has raised the question of how to deal with unexpected medically relevant information (Berg, Khoury & Evans, 2011). There was some but not complete concordance among specialists as to what information should be returned to patients (Green et al., 2012), and a global strategy for the categorization of genes and mutations has been proposed (Berg et al., 2012). From the socio-ethical standpoint there are few published data to inform this discussion (Wolf et al., 2012). Our interview-based study aimed to collect empirical data on the actual benefits and harms experienced by consumers who purchased the 23andMe Personal Genome Service^®^ that includes testing for three relatively common *BRCA* mutations. We report here the actual experiences of individuals who were faced with unexpected genetic information that has personal, medical, prognostic and family health consequences.

## Participants and Methods

The study was approved by the external, AAHRPP-accredited Ethical & Independent Review Services Institutional Review Board (E&I Review Document IRB-1-02.5). The protocol involved identifying within the 23andMe database *BRCA* mutation-positive customers who were at least 18 years of age, had consented to participate in research, and had chosen to view their results within the BRCA report. Before viewing their BRCA results, customers are encouraged to read written materials that provide information equivalent to that included in pre-test genetic counseling (Supplemental Information). Customers then can decide if they wish to see their results for these particular genes. Only individuals who had agreed to that step were invited to participate in this study. A control group of mutation-negative customers matched for age, sex and ancestry were also selected. Eligible participants were emailed an invitation that stated: “The study will involve a phone call about learning your results for specific tests: the three most common *BRCA* cancer mutations that predispose to the development of breast and ovarian cancer in females, and prostate cancer in males. You may or may not have one of these mutations”. Interested customers clicked on a button to learn more about the study and see the consent form. Those who agreed to participate were scheduled for a semi-structured phone interview by an experienced interviewer (CD) who did not know the *BRCA* status of the participants. Verbal consent was obtained from each research participant at the beginning of the phone interview.

Semi-structured Interview: During the interviews, we asked all participants whether they had been aware at the time of purchase that the 23andMe Personal Genome Service^®^ (PGS^®^) included tests for high-impact *BRCA* mutations. Further questions fell into several categories: personal cancer history, family cancer history, how long they waited to view their report and whether they recalled their results. Failure to recall the results of the *BRCA* report resulted in termination of the interview. Those who remembered their results were then asked another series of questions: a rating of emotional response across six possible categories (including measures of surprise and anxiety or relief), perception of personal risk for breast, ovarian or prostate cancer, who they had shared their results with, and what actions they had taken or were planning to take. Lastly, we asked participants to identify their ethnic background and provide a retrospective assessment of their experience of obtaining their *BRCA* results online. The interview guide was tested for understandability and clarity by engaging several employees of 23andMe and collecting feedback. The interviewer was able to ask follow-up questions to clarify the meaning of any specific answer. The interviews were taped, coded and transcribed. At the conclusion of the interviews, the BRCA mutation status of each participant was verified from the 23andMe database.

Data analysis: A total of 29 themes were identified from the topics covered in the interview by the interviewer and first author who had reviewed the transcripts. Two other authors (AKK and BM) then independently read through and coded each transcript for the presence or absence, and other qualitative or quantitative parameters, of each of the identified themes. Agreement between the coders was quite high, over 95%, for the majority of themes. Themes that had lower agreement were: anxiety, actions taken by female relatives, perceived benefits of PGS testing, and discussion with a medical profession. The coders re-reviewed the transcripts where there were specific areas of disagreement and then, through discussion, decided on a final coding. The majority of initial disagreements resulted from coder error, lack of clarity on how to code reactions or actions taken in response to prior BRCA testing, lack of clarity on how to code actions that were planned but not yet taken, and interpretation of qualified responses to the perceived benefits of the product.

## Results

### Study population

We identified 204 *BRCA1* (185delAG or 5382insC) or *BRCA2* 6174delT mutation carriers (130 males and 74 females) in the 23andMe database of 114,627 customers who were at least 18 years of age and had consented to participate in research. The male–female ratio reflects the gender distribution in the overall 23andMe customer base. Of the 204 mutation carriers, 136 (67%), 77 men (59%) and 59 women (80%), had viewed their *BRCA* report and were invited to participate in this study. In comparison, 65% of all customers in the database had viewed their *BRCA* report (63% of males and 67% of females). We selected a control group of customers who did not have one of the three *BRCA* mutations, matched to the mutation carriers by age, sex and ancestry. Five mutation-negative participants were unable to recall their *BRCA* results and therefore did not complete the full interview. Two rounds of recruitment were required to recruit a comparable number of mutation-negative individuals. The demographics of the final set of individuals who completed interviews (32 mutation-positive “cases” and 31 mutation-negative “controls”) are detailed in Table 1. While the control group included a few more females than males, the age range and mean age were very similar.

**Table 1:** Demographics and Awareness.

	Cases (*n* = 32)		Controls (*n* = 31)	
	Females	Males	Females	Males
Completed Interview	16	16	18	13
Age Range	30–73	26–62	27–73	23–66
Mean Age by Sex	51	43	50	43
Mean Age by Group	47		47	
When you purchased the 23andMe Personal Genome Service^®^ were you aware that it included testing for mutations that predispose to breast and ovarian cancer?				
Yes	7	6	10	10
No	9	10	8	3
Were you aware that having or not having Ashkenazi Jewish ancestry influences your risk of carrying one of the three mutations 23andMe is testing for?				
Yes	10	10	8	5
No	6	6	10	8

### Awareness of *BRCA* test and ancestry-based risk

At the time of purchase, only 13/32 (41%) cases were aware that *BRCA* testing was included in the PGS^®^, compared to 20/31 (65%) of controls (Table 1). In contrast, 20/32 (63%) cases were aware that having Ashkenazi Jewish ancestry influences the risk of having one of the three *BRCA* mutations, compared to 13/31 (42%) of controls.

### Personal and family history of cancer

Answers to the questions regarding personal and family history of cancer are summarized in Table 2. As expected, more cases than controls had a personal or family history of breast and ovarian cancer; there was little difference between cases and controls in terms of personal or family history of prostate and other cancers.

**Table 2:** Personal and Family History of Cancer.

		Cases (*n* = 32)		Controls (*n* = 31)	
		Females	Males	Females	Males
		(*n* = 16)	(*n* = 16)	(*n* = 18)	(*n* = 13)
When you purchased the 23andMe Personal Genome Service^®^ – had you been diagnosed previously with breast, ovarian or prostate cancer?					
Yes					
	BC	2		1	
	BC/OC	2			
	PC				1
No		12	16	17	12
Had you been diagnosed previously with any other cancer?					
Yes					
	Testicular		1		
	Melanoma		1		
	Sarcoma			1	
No		16	13	17	12
Had a first or second degree relative been diagnosed previously with breast or ovarian cancer?					
Yes					
	BC	10	9	6	6
	OC	6	5	1	
No		5	4	11	5
Had a first or second degree relative been diagnosed previously with any other cancer?					
Prostate		0	4	5	4
Pancreatic		1	2	1	
Colon		1	1		2
Gastric		1	1		1
Melanoma					2
Lung		2	1		
Other		uterine(1)	bladder(1)	esophageal(1)	leukemia(1)
					lymphoma(1)

**Notes.**

BC – breast cancer; OC – ovarian cancer; PC – prostate cancer.

### Viewing of the *BRCA* report

To avoid precipitating the discovery of a *BRCA* mutation in people who did not already know about their mutation status, we only invited customers to participate if they had chosen to view their *BRCA* report. In the current structure of the 23andMe results-reporting website, BRCA reports are “locked”, which means they require an additional customer approval step to “open” (display) the result. This feature enables customers to view other health reports before separately choosing to view the BRCA report. During the interview, we asked about their recollection of opening the report. Responses show that one case and nine controls did not remember *whether* the *BRCA* report required an extra step to open, and one case and five controls did not remember *when* they opened it. The majority, 24 cases (75%) and 18 controls (58%), however, remembered that they viewed it immediately, as soon as it was available to them (Table 3). Seven cases and eight controls waited for days to months before viewing their *BRCA* results. Reasons given for this delay included uncertainty about wanting to know, being too busy with other tasks, and not noticing the report until some later time.

**Table 3:** BRCA Test Results.

	Cases (*n* = 32)		Controls (*n* = 31)	
	Females	Males	Females	Males
	(*n* = 16)	(*n* = 16)	(*n* = 18)	(*n* = 13)
Was your BRCA report locked?				
Yes	14	15	11	9
No		1	1	1
Do not remember	1		6	3
When did you unlock it?				
Immediately	11	13	11	7
Later	4	3	6	2
Do not remember	1		1	4
What did you learn from your results?				
I learned for the first time that I am a carrier of a BRCA1 or BRCA2 mutation	11	14		
I had previous testing for these mutation and already knew that I am positive	5	2		
What was the reason for previous testing?				
I was diagnosed with breast or ovarian cancer	3			
A close female relative with breast or ovarian cancer tested positive for one of these mutations	1	1		
A close female relative with breast or ovarian cancer	1	1		
I learned for the first time that I am NOT a carrier of a BRCA1 or BRCA2 mutation			18	13

### Recollection of the *BRCA* test results

The blinded interviewer asked participants whether they remembered their *BRCA* results and what they learned from them. Five individuals did not remember their results and were excluded from further participation in the study. All of these were mutation-negative. Eleven women and 14 men stated that they learned for the first time that they had a mutation in either *BRCA1* (*n* = 10) or *BRCA2* (*n* = 15). Seven participants (five females and two males) knew prior to obtaining their results that they carried one of the three *BRCA* mutations that are tested for as part of the PGS^®^. Confirmation of their carrier status by the PGS^®^ increased their confidence in 23andMe test results and encouraged some to initiate testing of their relatives who they understood might also be carriers. Reasons for prior *BRCA* testing included a diagnosis of breast and/or ovarian cancer (three women), or having a first-degree relative with breast and/or ovarian cancer – with and without a known *BRCA* mutation (two men and two women) (Table 3).

All participants in the control group recalled their negative mutation status correctly. None of the mutation-negative individuals had been tested previously, they first learned this information through the PGS^®^ testing. Mutation status as recalled by each participant was independently confirmed through inspection of the database.

### Emotional responses to *BRCA* test results

Participants were asked about their initial emotional responses to seeing their 23andMe *BRCA* results. They were asked if they felt surprised by their results, and why or why not (Table 4). Ten mutation-positive individuals (six women and four men) who expressed surprise referenced the lack of a family history of breast or ovarian cancer or presence of only sporadic late-onset breast cancer, “not the genetic type”. Others were surprised because they believed the frequency of these mutations to be low in the general population.

**Table 4:** Responses to BRCA Test Results.

	Cases (*n* = 32)		Controls (*n* = 31)	
	Females	Males	Females	Males
	(*n* = 16)	(*n* = 16)	(*n* = 18)	(*n* = 13)
Were you surprised by this result?				
Yes	6	4	1	0
No	10	12	17	13
How did you feel about this information?				
Extremely upset (cried, lost sleep, had thoughts of suicide…)	0	0	0	0
Moderately upset (couldn’t stop thinking about the result, felt moderate anxiety)	3	1	0	0
Somewhat upset (initial disappointment, felt anxious at first but then anxiety went away)	3	6	0	0
Neutral	9	8	7	8
Relieved	1	1	10	5
Extremely relieved (if had high anxiety before)	0	0	1	0

Ten of the mutation-positive women were not surprised, including the five who had been previously tested, one who had close relatives known to be mutation-positive, and four others with a strong family history. The 12 mutation-positive men who were not surprised cited the following as reasons: two already knew about their mutation, one had a mutation-positive aunt, some had a positive family history of breast and/or ovarian cancer and others had realized they were at risk by having Ashkenazi Jewish ancestry.

Participants were read six options (which overlapped the Impact of Events Scale) and asked to choose which emotional response best fit how they felt at the time of receiving their results (Table 4). For the mutation-positive group, remarkably, none of the 32 reported having been “extremely upset”. Three of the four who were “moderately upset” were also “surprised” or “shocked” by discovering that they carried a *BRCA* mutation. The nine participants who said they had been “somewhat upset” initially, with anxiety subsequently subsiding, include three who were also “surprised”. Remarkably, nine women and eight men who were mutation positive reported feeling “neutral”. Of these 17, four women and two men already knew that they were *BRCA* mutation carriers. One female carrier was “relieved” to get confirmation of her previously known result. One young woman who was not “surprised” and felt “neutral” said:

*“I wasn’t surprised. It didn’t come as a surprise and, like I said, it wasn’t scary to me and it wasn’t bad news. It was just kind of more information to work with I guess. Yeah. I think I’m the only person in my family who’s had this testing so far. And I maybe would have different feelings about my results if, you know, I lost my mother to breast cancer, if it was a more emotionally charged thing, or if I knew somebody else who had it or something like that. I think it would maybe feel differently, but as far as I know I’m the only person whose been tested for it”.*

One male who learned about his carrier status for the first time reported feeling “relieved” because his daughter who was tested simultaneously by 23andMe had not inherited his mutation.

In the mutation-negative group, only one woman reported feeling “pleasantly surprised”; she also felt “extremely relieved” because she had a family history of breast, prostate and pancreatic cancer. The remaining 30 did not report feeling surprised, despite the fact that 12 had a family history of breast cancer in a first or second degree relative and one had a family history of ovarian cancer (Table 2). The emotional responses they identified most with were “neutral” or “relieved” (48% in each category), with 66% of the “relieved” responders being female (Table 4).

### Effect of *BRCA* test results on perception of cancer risk

We explored whether knowing one’s *BRCA* status affected the perception of one’s own breast and ovarian cancer risk (for women) or breast and prostate cancer risk (for men). Most female carriers reported understanding their personal risk as significantly elevated, and several correctly recited the risk figures that they had learned from their 23andMe report for both cancers. Some stated that their perception had not changed much because they had always believed they were at high risk due to breast/ovarian cancer in close family members and Ashkenazi Jewish ancestry, but that knowing they had inherited a *BRCA* mutation made them more aware of the reality and prompted them to take action. In general, male mutation carriers perceived their personal risk for breast cancer to be low and their risk for prostate cancer to be slightly higher than average.

The majority of *BRCA* mutation-negative participants stated that they perceived their cancer risk to be unchanged; only one woman thought her risk was reduced. The majority said they felt relieved not to be at high risk but realized that other genetic factors and environmental factors can still cause them to develop breast or ovarian cancer. Several women expressed the understanding that only the three common *BRCA* mutations were included in this test and that other mutations in these genes may still be present. Some mentioned that they had a slightly higher than average risk based on their separate 23andMe breast cancer report covering seven variants in other genes known to be associated with breast cancer, further conveying their understanding that there are many other risk factors to consider beyond BRCA mutations. Not a single mutation-negative participant interpreted the negative *BRCA* test result as indicating no or significantly lower than average risk for these cancers.

### Sharing of *BRCA* test results

We asked participants with whom they shared their *BRCA* results. The majority of mutation-positive participants shared their results with spouses/partners and blood relatives (Table 5). In addition, 50% (8 females, 8 males) shared their test results with friends, ranging from “a few friends” to “everybody”, which included using blogs or social media. Of the 32 mutation-positive participants, only two men had not yet shared it with anyone but said they were planning to share it with present or future spouses and other family members “as appropriate”.

**Table 5:** Sharing of BRCA Results.

	Cases (*n* = 32)		Controls (*n* = 31)	
	Females	Males	Females	Males
	(*n* = 16)	(*n* = 16)	(*n* = 18)	(*n* = 13)
With whom have you shared information about your 23andMe BRCA test results?				
Spouse	9	8	5	8
Other relatives				
Mother	4	6	5	3
Aunt(s)	5	3	2	
Sister(s)	7	7	4	2
Daughter(s)	3	3	1	
Father	1			
Brother			1	1
Son	3	1		
Cousin(s)	4	1		1
Niece(s)	1			
Grandmother				1
Stepmother		1	1	
Sister-in-law	1			
Friends	8	8	5	2
Primary care physician	4	5	5	2
OB/GYN physician	5		1	
Oncologist	4	1		
Genetic counselor	5		1	
Cancer geneticist	2			
Medical geneticist	1			
Other person		2		
Nobody		2	8	4

The majority of mutation-negative participants shared their *BRCA* results with spouses and family members, and some (7/31) shared with friends or medical care providers. The remaining 12/31 (39%) did not share their result with anyone, stating that they didn’t feel it to be relevant (Table 5).

### Communications with health care providers

Seven mutation-negative participants said they informed their primary care physician. Thirteen of the 16 mutation-positive women (including two who had previously tested positive) sought medical advice (most did so immediately) from their primary care physician, gynecologist or oncologist. Five consulted with a genetic counselor, usually upon referral from the primary care physician, who then coordinated repeat *BRCA* testing in a clinical lab (in most but not all cases) and referred them to an oncologist.

Overall, our interviews revealed that sharing of *BRCA* test results with physicians was more common amongst mutation-positive individuals (19/32 or 60%) than mutation-negative ones (8/31 or 26%), and within the mutation-positive group, it was also more common amongst women (13/16 or 81%) than men (6/16 or 38%). The three women who didn’t contact a physician when they got their result already knew that they were BRCA mutation-positive and had been treated for breast and/or ovarian cancer.

The rate at which mutation-negative participants shared their results with physicians is comparable to those reported in larger surveys of DTC customers: 27% (Bloss, Schork & Topol, 2011) and 20% (Kaufman et al., 2012). The high level of sharing with physicians for the mutation-positive women most likely reflects the actionability of the test results.

Some female mutation-carriers expressed feeling pressured into surgical procedures by physicians, and some stated that the full range of choices presented to them by genetic counselors had caused anxiety.

*“It was a real shake up for me for a little while. But not because of my reaction to the BRCA2 results from 23andMe, but to the total fear factor that was put in by all of the traditional medical people; based on my doctor telling me if I didn’t get my breasts and my whole female organs out within 6 weeks, and by the way that date has long since passed.”*

*“At the beginning I was not anxious. It was very rational, you know, it is what it is. Later I had slight anxiety, because there are so many choices. So if it had been like so okay, this is, you know, one way to do the surgery, that’s fine. But then I went to the genetic counselor, it’s like so many choices, and whenever you have choices you have anxiety, because it’s time to research and make right decisions and so on.”*

Although only a few mutation-negative females shared their results with physicians, those that did so felt their physician showed little interest, did not know what to do with the information or doubted the validity.

*“I gave a full printout of my results to my primary care physician, just for the heck of it. But it really didn’t come up in conversation. I didn’t really talk about it. I didn’t think I was at risk because it confirmed like, oh, I’m not at risk and that was that.”*

*“I enabled my surgeon and my internist both to have access to the information that I received from 23andMe. But I think essentially their feeling was that it wasn’t really helpful you know that it was purely a survey. It didn’t provide the type of genetic information that they would find extremely helpful.”*

*“I shared the results with my physician when I did have the mammogram. She kind of looked at me like physicians don’t know how to handle this information because that’s not part of their routine. So I don’t know if she took it in and goes – okay I don’t know what to do with this, I’m gonna go with what I know [that] is order a mammogram.”*

*“And I was telling my doctor about this. And I said I had this analysis and it showed different likelihood of this and that. I didn’t mention the breast one because it was never a concern. And he said how much did it cost, and I said I think it was $150 a year or something like that. And he said ‘Well, how can they have good results for only that much?’ And I said they do. They really do a very careful analysis and they’re constantly bringing new results. So he wants to see it sometime, so I’ll have to print it out because he’s open to it”.*

### Actions taken or planned in response to *BRCA* report

Mutation-positive participants were asked about the immediate next steps they took after learning their results and what they were planning to do in the future (Table 6). In terms of immediate action, sharing results with family and friends was the most common response. The majority of women also sought medical advice. Prior to being tested by 23andMe, three women had been diagnosed with breast cancer and had undergone mastectomies and one had also undergone a prophylactic oophorectomy. There were 11 mutation-positive women who received this information through 23andMe for the first time. Among these 11 women the following actions were taken or are planned: one prophylactic mastectomy, three planned mastectomies, three oophorectomies, and four planned oophorectomies (after childbearing). Five said they went to have breast exams and breast imaging after getting their results, and the seven who neither had nor were planning to have mastectomies reported that they would continue to have regular breast cancer monitoring (Table 6).

**Table 6:** Taking Action After Positive 23andMe BRCA Result.

	Cases (*n* = 32)	
	Females	Males
	(*n* = 16)	(*n* = 16)
What did you do next?		
Talk to family/friends	7	7
Consult my primary care doctor	4	1
Consult a genetic counselor	3	
Consult a cancer specialist (OB/GYN, oncologist)	6	
Consult a cancer geneticist	2	
Repeat BRCA testing in a clinical lab	3	2
Had physical breast/ovarian exam and breast imaging	5	
Had risk-reducing mastectomy	1	
Had risk-reducing oophorectomy (removal of ovaries)	2	
Did online research	1	2
Quit smoking, changed diet		1
What are you planning to do in the future?		
Will have regular breast/prostate cancer surveillance screening	7	13
Will have risk-reducing mastectomy	3	
Will have risk-reducing oophorectomy	4	
Will make sure my mother/sisters/daughters/brothers/sons get tested	12	5
Will mention BRCA results to physicians	2	
Will ask oncologist for prophylactic drug treatment	1	
Will recommit to staying healthy	1	
Will stay updated on breast cancer research	1	

In general, male carriers did not initially consult a physician; several stated that they were familiar with the literature, had done online research and/or were working as professionals in the field and did not feel they needed medical advice. The majority, 13/16 (81%), said they would seek or would continue to have regular breast and prostate cancer screening.

*“It’s a little bit interesting as a male approaching this, because I can’t quite put myself in the female perspective. But the combinations of prophylactic type procedures that are available for a female with these results are very, very different than what’s available to me. So, no, I don’t have anything directly planned other than just to be aware.”*

*“Breast cancer, yeah, fairly low. I know it’s possible, but it’s not particularly common. I mean, I don’t know if the studies have been linked strongly with prostate cancer, but it follows in my family directly. So that’s the one I’ve been thinking about.”*

*“I might start my prostate cancer screening earlier, maybe at 45 or 40 even. I might tell my physician I have this gene and that it might be a good idea to get tested younger than the recommended age.”*

The present study reveals that for those who received a positive *BRCA* test result from 23andMe, there was a distinctly positive effect of identifying additional family members at risk.

We observed a significant expansion in identification of *BRCA* carriers through testing of relatives of both female and male mutation carriers.

Male carrier: “*My mother saw a genetic counselor as a result of my testing and my little sister saw a genetic counselor as a result of my test.”*

At the time of our interviews, 30 secondary (family member) *BRCA* tests had been carried out as a result of the initial testing, either through physician channels or through 23andMe, with 13 positive and 17 negative results. In one of these mutation-positive secondary cases, early breast cancer (DCIS) was discovered by MRI, a sensitive imaging procedure usually offered as a screening test only to mutation-positive women (Robson & Offit, 2007). Several secondary cases with mutation-positive BRCA tests also underwent prophylactic mastectomy and oophorectomy and others indicated that they were planning to have both risk-reducing procedures in the future.

### In retrospect: Perceived benefits outweigh harms

At the end of the interview, we asked participants whether they thought purchasing the PGS^®^ was worth it, whether they would do it again, and why or why not. 30/32 cases and 30/31 controls said they would do it again. The participants found the PGS^®^ worthwhile for several reasons; the most significant being that some mutation carriers felt it may have saved their lives or the lives of relatives who tested *BRCA* positive as a result of the primary participant being tested. In addition, participants received health information unrelated to BRCA that they found useful for personal risk assessments or family planning, or thought the PGS was worthwhile because they discovered or confirmed their ancestry, or just had their curiosity satisfied.

### Positive impacts

Female carrier: “*While the results were shocking and a little stressful, ultimately I think this could potentially change my life, and it obviously made a difference for my aunt, who was able to catch pre-cancer early. So I think all in all, it’s a positive thing. We would have never known, because there are no [affected] first and second-degree relatives. So until somebody ended up with breast or ovarian cancer I don’t think we would have known. This way we’re taking care of things prophylacticly.”*

Female carrier: “*Well, I got information that I could do something about. That’s what I’m telling people. You know, if you get information that you can do something about, and I think particularly Ashkenazi Jewish women, it’s certainly worth doing. I mean if someone did say to me ‘I don’t know what I would do with the information if I had that information,’ but I’m a proactive person, so it’s hard to imagine someone not doing something.”*

Male carrier: “*Even after I talked with genetic counselors, they never would have recommended me to be tested based on my family history that I knew of at the time. I feel like a genetic secret was found in my family. I feel like I may have saved my sister’s life. I mean, nobody knows. That’s a hard thing in our family because nobody had cancer early, but it makes a difference. You accept it. It’s in your system and I’m lucky enough to know that it’s there as opposed to finding out something too late. It increases your odds you’re going to find something and find it earlier.”*

Male carrier: “*I immediately had my children tested, finding that my daughter was predisposed (sic) to BRCA1 and she’s now being treated because of this. In other words, 23andMe may very well have saved my daughter’s life. My doctor and we have spoken to many, many of our friends to tell them about what we think about 23andMe. It’s all positive. I bet we’ve told 150 people.”*

Male carrier: “*Well, first off, specifically for this issue of the BRCA thing, it provided me information. We had cancer in the family. We had reason to wonder and it solved it very clearly relating to my daughter. I don’t think that learning that I have a few percentile or lower risk of anything is going to change my life, but some things are interesting, some things are informative and I think it’s important.”*

Male carrier: “*I think it’s good to know these things, regardless of whether it’s something you can control or do something about. In the case of this particular mutation, there’s only a 50% chance that if we had a daughter she would inherit the same gene. And maybe I would want – knowing now that I am a carrier – I would want, if we have a daughter, I would want her to get genetic screening. I think it’s a good thing.”*

Male carrier: “*And as healthcare becomes more and more streamlined and more specific, or I don’t know exactly what the word I’m looking for there is, but as it becomes more treatment focused that you need to be aware as possible of what your health situation is and what your risks are, because you can’t expect the healthcare provider to have all that information. You need to have your information, as much information as possible to make good decisions.”*

### Negative impacts

Only one of the 63 participants reported a negative impact. This participant was a mutation-positive man with a family history of breast cancer. He reported “emotional cost” and said he would prefer not to know his mutation status.

*“I would not do it again, because it is really not information I need to know. I don’t think the cost in dollars was important, I think the emotional cost is more. The impact of the results to a greater extent was negative. It’s just basically knowing that I have it, I might pass it on. And that’s the main thing. Sometimes ignorance is better.”*

In addition, one other case and one control said they would not purchase the 23andMe PGS^®^ again. One woman already knew that she was positive for the *BRCA* mutation and had had a double mastectomy in the past; therefore, she felt that her PGS^®^ result did not provide her new information. The mutation-negative participant who would not purchase the PGS again had received the kit as a gift and reported prior emotional instability as a reason for her preferring not to know:

*“I guess denial is a pretty powerful feeling, and sometimes not knowing something helps you just forage through life without having some details that might cloud the way you perceive things. And so I guess a little bit of rosy-colored glasses would help someone like myself. I guess I don’t want to think negatively. Even if it were the truth, I might choose not to know that.”*

### Impact on male carriers

Many male carriers expressed strong concern about the risk of passing on a *BRCA* mutation to their daughters. Despite the fact that female carriers have the same risk of passing on the mutation, they did not mention this as a major concern since they were primarily focused on their own risk for disease and on making decisions about risk-reducing procedures.

Interview quotes from male carriers:

*“I was never concerned for myself, even though when I found out, the first thing that came to my mind was: will my daughter carry this. So I was extremely concerned but not for me, if I answer extremely concerned, it wasn’t for me at all, but if you’re asking what my emotion was it was extreme concern, but not over my own situation.”*

*“I do see that I have a little chance of getting breast cancer even though I’m a male, and it’s probably increased in males with BRCA1 compared to normal males, but that is a very low chance. So I’m not worried about myself. I might be a little worried if I get married and have children, because it has 50% chance of passing on, and if the child is a female then she has a high risk…. initially my thought was I shouldn’t have children.”*

*“For breast cancer, I guess being a male the statistics are not so dramatic about that, what I read on your site about it. So as far as my health is concerned, and I can also compare to my father and grandfather who must have had the mutation, I’m at some risk to die sometime from something but it’s not really an anxiety factor. I would have been alarmed had I learned that my daughter had it, but I guess luckily she doesn’t.*”

### Comprehension of report

We scrutinized the interview transcripts for any evidence of misunderstanding of the information provided in the online *BRCA* report. One female mutation carrier was under the impression that all 23andMe health reports deal solely with disease probabilities, and did not appreciate that *BRCA* mutation testing provides a definitive answer about carrier status.

*“I really read it as what it basically is. It’s not that I necessarily am a carrier. I’m just you know statistically it’s likely that I could be.”* This participant was also confused about the mode of inheritance: *“I have 5 children and 3 of them are daughters. Well it doesn’t matter because it’s gonna be passed through the boy.”*

She had described her initial response as “surprised”, because her mother who had breast cancer was not Jewish, and her emotional reaction as “neutral”. She subsequently consulted with her physician and received post-test genetic counseling which she described as extremely upsetting and which did not alleviate her misunderstanding.

Two mutation-positive participants interpreted the fact that the *BRCA* report was initially “locked”, requiring an extra click to opt-in to view the report, as indicating a positive result:

“*When you put a locked result on and you say this is locked, you don’t have to open it, well, everybody knows what that means. As soon as you say this result is locked and you can click here if you want to open it, you know it’s going to say something not good.”*

*“As soon as I saw it was locked, I opened it right away ‘cause I knew what it was. I mean there was no reason it was gonna be locked unless it was a mutation.”*

There was no evidence among the mutation-negative group that any of them interpreted their data to suggest a significant reduction in breast cancer risk. All but one reported their perception of their own risk for breast and ovarian cancer to be unchanged after learning about their *BRCA* mutation-negative status.

## Discussion

The data reported here clearly demonstrate that providing *BRCA* mutation results directly to consumers benefited a large fraction of individuals. A number of women without a family history of early onset breast or ovarian cancer discovered that they carry a *BRCA1* or *BRCA2* mutation that conveys a high risk. In many families, these benefits were extended to relatives of male or female mutation-positive study participants who took the initiative to be screened after learning of the 23andMe customer’s result and tested positive. Newfound awareness of their high risk led the majority of these women to seek medical advice and many took or are planning to take risk-reducing actions upon the advice of their physicians.

None of our 32 mutation-carrying study participants reported serious emotional disturbance such as feeling extremely upset, suicidal or requiring professional psychiatric help after learning of their mutation status. Four mutation carriers reported that they were moderately upset and nine reported transient anxiety after learning their mutation status. Emotional distress shortly after learning significant genetic information is not unexpected, but decreases over time, as reported in a meta-analysis of women who had undergone *BRCA* mutation testing within the healthcare system (Hamilton, Lobel & Moyer, 2009). There is greater concern over emotional disturbance that is more severe and lasts for weeks, months or years. We did not obtain reports of long-term emotional disturbance. Only one of 32 mutation-positive individuals, a male, regretted obtaining his mutation status. In contrast to expectations, half of the mutation-positive participants of either gender described their emotional response as neutral.

Our results are comparable to those of a study on the disclosure of *APOE* genotypes to adult children of patients with Alzheimer disease (AD) that measured symptoms of anxiety, depression and test-related distress (Green et al. REVEAL study, 2009). In contrast to our study of DTC customers who received unexpected results, the REVEAL study compared disclosure and nondisclosure groups of participants (that both were pre-screened and underwent extensive pre-test counseling and post-test psychological follow up) and failed to find significant differences in test-related distress. This is even more remarkable given the current lack of preventive measures for AD in contrast to the preventive options available to *BRCA1* or *BRCA2* mutation carriers.

The majority of participants shared their *BRCA* results with family and friends, and all female carriers consulted with health care providers and were guided to appropriate actions such as more frequent monitoring or risk-reducing mastectomy and/or salpingo-oophorectomy. A large prospective multi-center cohort study has demonstrated the effectiveness of these procedures in BRCA mutation carriers for lowering the risk of breast and ovarian cancer and reducing cancer-specific mortality and all-cause mortality (Domchek et al., 2010).

Female mutation-positive participants with a personal or family history of breast/ovarian cancer were more aware of their risk prior to 23andMe testing, and five had received BRACAnalysis^®^ testing previously. In contrast, where the primary mutation-positive individual identified through 23andMe was male or the mutation was transmitted through the male line, there usually was no family history of breast/ovarian cancer and the identification of a *BRCA* mutation came as a complete surprise. The female members of these families were not eligible for *BRCA* testing within the healthcare system and, in the absence of population-wide screening for these mutations, they would not have known about their increased cancer risk. We do not know the final tally of first- and second-degree relatives who were tested because of the primary customers’ positive *BRCA* test results; 30 were known at the time of the interviews. Several of the 13 secondary cases who tested positive had undergone or planned to have risk-reducing surgical procedures. Major effects on family members were also reported for the single case studied by Dohany et al. (2012). A population-based study from Denmark reported on uptake and timing of risk-reducing surgery in 306 healthy *BRCA* carriers with no personal history of ovarian and breast cancer. The 10-year uptake was 75% for salpingo-oophorectomy and 50% for mastectomy (Skytte et al., 2010). In a prospective study conducted in Washington DC, five years after *BRCA* testing more than 80% of mutation carriers had obtained one or both risk-reducing procedures (Schwartz et al., 2012). It is worth noting that for younger women the discovery of a BRCA mutation can lead to difficult decisions about considering risk-reducing surgery in the face of uncertainties about the effectiveness of screening procedures (MRI versus mammography) and can cause considerable anxiety.

While some mutation-positive female customers crusaded within their extended families with missionary zeal, others, most often males, felt a considerable burden at having to inform their at-risk relatives. One was distressed after being told by a genetic counselor that informing relatives was his duty, and another asked us for a guidance document that would tell him how best to convey that information. In a systematic review of qualitative studies on communication of inherited cancer risk to at-risk relatives, Chivers et al. (2010) identified themes that facilitate or inhibit such interactions, such as tension between the duty to inform and the wish not to harm or distress the recipients of the information; for *BRCA* mutations, specifically, carriers were uncertain about whether and how to inform male relatives and they desired health professionals’ input. The burden of the role of informant has been well illustrated by the bioinformatician Manuel Corpas who was managing the 23andMe accounts of his extended family (Corpas, 2011; Corpas, 2012).

While male *BRCA* carriers responded positively by adhering to recommended prostate cancer surveillance or starting it at an earlier age, the 50% chance of passing on the mutation to their children caused many of them considerable emotional distress. In current medical practice, children under age 18 are not tested for adult-onset genetic diseases that lack preventive treatment options during childhood. 23andMe enables the testing of children, with parents becoming the custodian of the information. As articulated by several study participants, this opportunity to have their children tested shifted their burden from anxiety over not knowing whether they passed on the mutation to relief if they didn’t, or if they did, to facing the decision of when and how to inform their carrier children.

One indirect way that we assessed the extent of harm to participants who chose to view their *BRCA* test results was by asking them if they would purchase the service again. Only one female non-carrier, who had obtained the PGS^®^ as a gift, and one male carrier, stated that they would prefer not to know their result. The male cited distress about genetic risk for his future daughters as the reason. A previous small study of predictive testing for *BRCA* mutations in males from mutation-positive breast cancer families reported that concern about transmitting increased cancer risk to daughters was the major motivating factor for testing. In that study, four of 18 males who tested positive had adverse responses, with three refusing to disclose the test results to their family and one regretting his participation (Daly et al., 2003). In another study, none of the parents, including men, found it difficult to inform their adult children of their *BRCA* mutation (Hallowell et al., 2005).

With respect to misunderstanding *BRCA* test results, we identified one female participant who reported confusion about the meaning; she had also sought genetic counseling, which had not relieved her confusion – in fact, she described it as having added to her distress.

Remarkably, none of the male mutation carriers interpreted their results as having no relevance to their female relatives. The *BRCA* report in the 23andMe PGS clearly states: “*Their own risk aside, it is important for men to know whether they are carriers of BRCA mutations because they have a 50% chance of passing the mutation on to their daughters, who would then be at increased risk for breast and ovarian cancer. The mothers and sisters of men who carry one of these mutations also have a 50% chance of being carriers*”. In contrast, the literature suggests widespread misperception about paternal transmission of breast cancer risk. Many women and their physicians underestimated the significance of a family history of breast cancer on the father’s side of the family (Metcalfe et al., 2010). In that study, only 34% of 273 women with early-onset breast cancer knew that a father can pass an abnormal breast cancer gene to his daughter, comparable to 29% of 628 Swiss physicians surveyed by Pichert et al. (2003). 23andMe regularly updates its reports in order to maximize the chance that customers understand those reports and the concepts underlying those reports. Although genetics education is a mission of a broad array of stakeholders, DTC personal genomics companies are in a unique position to provide such educational materials. In our experience, consumers appear more likely to read and learn about an unfamiliar topic such as genetics when the information is personalized. The responses of physicians to the BRCA DTC results were distinctly different for women with positive and negative results. Physicians reacted to positive results with appropriate referrals to genetic counselors, requesting confirmation of BRCA results in another lab (all of those were confirmed) and referrals to oncologists for treatment. Few of the BRCA-negative individuals shared their BRCA reports with physicians and found them unhelpful; their physicians did not know what to do with the reports and did not appreciate the value of the information.

Our study has several limitations. In conducting it, we relied on volunteers willing to share their experiences. The emotional responses reported by the 16 men and 16 women who are BRCA carriers and agreed to be interviewed for this study may differ from those experienced by the 61 male and 43 female mutation – positive individuals who did not reply to our invitation. Customers of 23andMe are also not entirely representative of the overall population, and the participants in this study may represent a pro-active more scientifically educated group, although no data on educational background were collected as part of this study. We cannot exclude that somebody who had a traumatic response may not have wished to revisit that experience in an interview. However, the initial study participation rate was higher within the mutation-positive group than within the mutation-negative group. In addition, not a single instance of serious adverse reaction has been brought to our attention either through this project or other projects. The case reported by Dohany et al. (2012) represents a customer who initially experienced distress and successfully made use of the post-test genetic counseling that is recommended in all 23andMe reports for those who may require it. Furthermore, as the time interval between viewing their *BRCA* report and the interview varied between two years and three months, the accuracy of recollections may have differed among participants.

Current practice guidelines, and insurance reimbursement, limit *BRCA* mutation testing to women with early onset breast or ovarian cancer and their first degree female relatives, as well as to close relatives of mutation-positive index cases (Robson, 2003). This policy missed the *BRCA* mutation status of Jill Steinberg, whose sister and mother had early onset breast cancer but were *BRCA* mutation negative. Jill discovered her mutation through 23andme PGS^®^ and found that she had inherited it from her father who had prostate cancer (Saunders, Nazareth & Pressman, 2011; Steinberg, 2011).

When unselected Jewish women in Canada were screened for these three founder mutations, the mutation prevalence was reported as 0.5% for *BRCA1* and 0.6% for *BRCA2* (Metcalfe et al., 2010). Within two years after learning of their BRCA mutation, 11.1% of women had undergone prophylactic mastectomy and 89.5% prophylactic oophorectomy; and those who had these procedures reported reduced distress (Metcalfe et al., 2012). Given the frequency of these three mutations in the Ashkenazi Jewish population, a population-wide screening program has been proposed (Metcalfe et al., 2012). When considering the target population for such a screening program, it is important to realize that in our study six cases and six controls self-identified as non-Jewish, and one control as Jewish non-Ashkenazi (Table S1). One of the controls was adopted with unknown biological parents. The other cases and controls who identified as Caucasian non-Jewish reported Eastern European ancestry, except for one mutation carrier who self-identified as East Indian. His *BRCA1* 185delAG mutation has been previously identified in breast cancer patients in India (Vaidyanathan et al., 2009), as well as in breast/ovarian cancer families with Sephardic Jewish ancestry in Spain (Díez et al., 1999) and in descendents of early Spanish settlers in Colorado (Mullineaux et al., 2003). Therefore, limitation of testing for these three mutations to people who identify as Ashkenazi, could miss *BRCA1* 185delAG carriers with Sephardic Jewish ancestry. On the other hand, *BRCA1* or *BRCA2* mutations besides the three most common ones have also been reported in Ashkenazi Jewish women with breast/ovarian cancer although at much lower frequencies (Kauff et al., 2002).

Outcome studies of relatives of *BRCA* mutation carriers provided strong evidence that carrier identification is beneficial with high uptake of risk-reducing procedures and reduced cancer risk and cancer-specific mortality (Skytte et al., 2010; Domchek et al., 2010). Given the high rates of appropriate medical follow-up for the individuals and their family members, the absence of significant emotional distress and reports of inappropriate follow-up, and the ethnic diversity of the mutation carriers, our data support the establishment of population-based screening programs for these three relatively common mutations.

With the frequency of genome/exome sequencing studies increasing rapidly, the discussion of how to deal with unexpected medically relevant data is currently at the forefront (Berg, Khoury & Evans, 2011; Berg et al., 2012; Green et al., 2012), with little published empiric data to guide it (Wolf et al., 2012). Most of the information that is currently provided by whole genome/exome studies involves low-impact variants affecting the risk of common diseases or variants of unknown significance. In contrast, BRCA mutations represent the group of highly penetrant autosomal dominant neoplastic disorders with available management options. Incidental discovery of such mutations engenders a different set of responsibilites and difficult choices for patients and their relatives. In our study, 25 individuals who did not know they were carrying a BRCA mutation were given unexpected and potentially life-saving information and 30 additional relatives were screened, with an added discovery of 13 positive cases. Therefore, the experiences of these individuals who were faced with unexpected genetic information that has personal medical, prognostic and family health consequences and their responses to the information, how they dealt with it emotionally and practically, and their relatives’ willingness to learn about their risks, will be useful in informing this discussion.

## Supplemental Information

10.7717/peerj.8/supp-1 Supplemental Information 1

10.7717/peerj.8/supp-2 Supplemental Information 2 Supplemental Data From 23andMe BRCA Report: Information for customers to help them decide whether or not they wish to see their data
