# Supplementary material for: Dealing with the unexpected: consumer responses to direct-access BRCA mutation testing
Source: PeerJ. 2013 Feb 12;1:e8. doi: 10.7717/peerj.8 (PMC3628894; doi:10.7717/peerj.8)
Supplement: Supplemental Information 1 [file peerj-01-8-s001.docx]

| **Table S1. Self-declared ethnicity** | | | | | | |
| --- | --- | --- | --- | --- | --- | --- |
|  | **Cases (n=32)** | | | **Controls (n=31)** | | |
|  | Females  (n=16) | | Males  (n=16) | Females  (n=18) | | Males  (n=13) |
| **What is your ethnic background?** | | | | | | |
| Ashkenazi Jewish 100% | 9 | 7 | | 8 | 7 | |
| Ashkenazi Jewish >50% | 1 | 2 | | 5 | 1 | |
| Ashkenazi Jewish 50% | 2 | 2 | | 1 | 0 | |
| Ashkenazi Jewish <50% | 0 | 2 | | 1 | 0 | |
| Caucasian (White), non-Jewish | 3 | 2 | | 2 | 2 | |
| Hispanic/Caucasian | 0 | 0 | | 0 | 1 | |
| Jewish, not Ashkenazi | 0 | 0 | | 0 | 1 | |
| Asian (Indian) | 0 | 1 | | 1 | 0 | |
| Unsure | 1 | 0 | | 0 | 1 | |
