# Supplementary material for: Dealing with the unexpected: consumer responses to direct-access BRCA mutation testing
Source: PeerJ. 2013 Feb 12;1:e8. doi: 10.7717/peerj.8 (PMC3628894; doi:10.7717/peerj.8)
Supplement: Supplemental Information 2 — From 23andMe BRCA Report: Information for customers to help them decide whether or not they wish to see their data [file peerj-01-8-s002.docx]

**Supplemental Data**

From 23andMe BRCA Report:

Information for customers to help them decide whether or not they wish to see their data

Your data and results do not affect whether you see the text below. Everyone must view this information and explicitly unlock the report to see their results.

**Before you view your data...**

Consider the following before you view your genetic data regarding Breast/Ovarian Cancer:

- **The influence of environmental factors:** The risk for breast and ovarian cancer is only partially determined by genetics. Environmental factors, including but not limited to diet and lifestyle, also play significant roles.
- **This is not the entire genetic picture:** The mutations reported by 23andMe account for only a portion of the entire genetic contribution to breast and ovarian cancer. There are other known mutations, including many in BRCA1 and BRCA2, for which 23andMe does not provide data. If you are concerned about these, you should consult a medical professional about taking specific tests that offer a more complete assessment of these two genes. There are also unidentified genetic factors that affect breast cancer risk.
- **Your ancestry affects your chances of having these mutations:** Though extremely rare in the general population, these mutations are much more common in families with Ashkenazi Jewish ancestry.
- **The mutations described here cannot predict definitively whether you will develop breast or ovarian cancer:** Though having these mutations greatly increases the risk for both diseases, many people who have them will never get the disease. Conversely, lacking these mutations does not substantially reduce your breast or ovarian cancer risk.
- **These mutations are also relevant to men:** Although men are not at risk for ovarian cancer and are at very low risk for breast cancer, BRCA1 and BRCA2 mutations can increase a man's risk for prostate cancer and male breast cancer. Men who carry one of these mutations have a 50% chance of passing it on to their daughters, who would then be at increased risk for breast and ovarian cancer. The mothers and sisters of men who carry one of these mutations also have a 50% chance of being carriers.

**I understand, please show results for:**[Name]
